# IL-25 exacerbates autoimmune aortitis in IL-1 receptor antagonist-deficient mice

**DOI:** 10.1038/s41598-019-53633-0

**Published:** 2019-11-19

**Authors:** Takamichi Yoshizaki, Satoshi Itoh, Sachiko Yamaguchi, Takafumi Numata, Aya Nambu, Naoyuki Kimura, Hajime Suto, Ko Okumura, Katsuko Sudo, Atsushi Yamaguchi, Susumu Nakae

**Affiliations:** 1 Laboratory of Systems Biology, Center for Experimental Medicine and Systems Biology, The Institute of Medical Science, The University of Tokyo, Tokyo, 108-8639 Japan; 2 Department of Cardiovascular Surgery, Saitama Medical Center, Jichi Medical University, Saitama, 330-8503 Japan; 3 Department of Dermatology, Tokyo Medical University, Tokyo, 160-0023 Japan; 4 Atopy Research Center, Juntendo University School of Medicine, Tokyo, 113-8412 Japan; 5 Animal Research Center, Tokyo Medical University, Tokyo, 160-8402 Japan; 6 Precursory Research for Embryonic Science and Technology (PRESTO), Japan Science and Technology Agency, Saitama, 332-0012 Japan

**Keywords:** Interleukins, Cardiovascular diseases

## Abstract

IL-25, a member of the IL-17 family of cytokines, is known to enhance type 2 immune responses, but suppress type 3 (IL-17A)-mediated immune responses. Mice deficient in IL-1 receptor antagonist (*Il1rn*^−/−^ mice) have excessive IL-1 signaling, resulting in spontaneous development of IL-1–, TNF– and IL-17A–dependent aortitis. We found that expression of *II25* mRNA was increased in the aortae of *Il1rn*^−/−^ mice, suggesting that IL-25 may suppress development of IL-1–, TNF– and IL-17A–dependent aortitis in *Il1rn*^−/−^ mice by inhibiting type 3-mediated immune responses. However, we unexpectedly found that *Il25*^−/−^*Il1rn*^−/−^ mice showed attenuated development of aortitis, accompanied by reduced accumulation of inflammatory cells such as dendritic cells, macrophages and neutrophils and reduced mRNA expression of *Il17a* and *Tnfa*—but not *Il4* or *Il13*—in local lesions compared with *Il1rn*^−/−^ mice. Tissue–, but not immune cell–, derived IL-25 was crucial for development of aortitis. IL-25 enhanced IL-1β and TNF production by IL-25 receptor–expressing dendritic cells and macrophages, respectively, at inflammatory sites of aortae of *Il1rn*^−/−^ mice, contributing to exacerbation of development of IL-1–, TNF– and IL-17A–dependent aortitis in those mice. Our findings suggest that neutralization of IL-25 may be a potential therapeutic target for aortitis.

## Introduction

Giant cell arteritis (GCA) and Takayasu arteritis (TAK) are the most common large-vessel vasculitides (LVV). They are infrequent, but potentially fatal, diseases. The pathologic hallmarks of LVV are focal granulomatous vasculitides, characterized by lymphomonocytic infiltration and occasional multinucleate giant cells^1^. Those lesions lead to intimal hyperplasia that predisposes to luminal obstruction, arterial wall remodeling with disruption of the elastic *laminae*, excessive deposition of extracellular matrix, and fibrosis^2^. Consequently, vasculitis involving the aorta, subclavian-axillary bed, carotid branches and arteries of all sizes can lead to vision loss, aortic arch syndrome, aortic dissection, and aortic aneurysms and rupture^3^. Although glucocorticoids are very effective for treatment of both GCA and TAK, relapse is common following reduction of the dose^4,5^. In such cases, methotrexate, an immunosuppressive drug, is used for sparing of glucocorticoids while preventing relapse^6–8^. Newer biologics, such as neutralizing antibodies for TNF-α, IL-6, and/or IL-12/IL-23p40, have also been developed for GCA and TAK^9^. Anti-TNF-α agents are effective for treatment of TAK, but not GCA^10–14^. Increased expression of IL-1β was observed in specimens from patients with LVV such as TAK^15^ and GCA,^16,17^ suggesting that this cytokine is responsible for the development of such diseases. In support of that notion, associations were reported between polymorphisms of IL-1B and IL-1RN genes and susceptibility to TAK^18^ and GCA^19^ in certain populations. In addition, mice deficient in IL-1 receptor antagonist (*Il1rn*^−/−^ mice) had excessive IL-1 signaling, resulting in spontaneous development of aortitis accompanied by infiltration of predominantly lymphocytes, which contributed to destruction of the elastic laminae with fibrosis, resembling LVV such as TAK and GCA^20,21^. The spontaneous development of aortitis in those *Il1rn*^−/−^ mice was dependent on T cells, whereas it was suppressed by deficiency of TNF-α or IL-17A, but not IL-6^22,23^. Based on those findings in *Il1rn*^−/−^ mice, a therapeutic trial of Anakinra (recombinant human IL-1RN) in GCA patients found that its blockade of IL-1 activity was effective for treatment of the disease^24^.

Using that same *Il1rn*^−/−^ mouse model, we found that the expression level of IL-25 (also called IL-17E), which is a member of the IL-17 family of cytokines, was increased in the aorta. IL-25 binds to IL-25 receptor (IL-25R), which consists of IL-17RA and IL-17RB. IL-25 is preferentially produced by epithelial cells and such immune cells as macrophages, mast cells, basophils, eosinophils and T cells^25,26^. IL-25 induces production of type 2 cytokines by various types of cells, including Th2 cells, Th9 cells, invariant NKT cells and group 2 innate lymphoid cells^27,28^. Those cytokines are involved in such type 2 immune responses as protection from nematode infection and development of allergic disorders^26–29^. In addition, IL-25 plays dual roles in type 3 immune responses: it can suppress IL-17A–mediated autoimmune diseases^25,30,31^, but it enhances IL-17A–mediated dermatitis^32,33^. However, the role of IL-25 in IL-1–, TNF– and IL-17A–mediated aortitis in *Il1rn*^−/−^ mice has been unclear. Here, we demonstrate that IL-25 plays a facilitative role in the development of IL-1–, TNF– and IL-17A–mediated aortitis in *Il1rn*^−/−^ mice.

## Results

### Identification of IL-1β– and IL-1Ra–producing cells in lesions of aortitis

**Figure 1:**
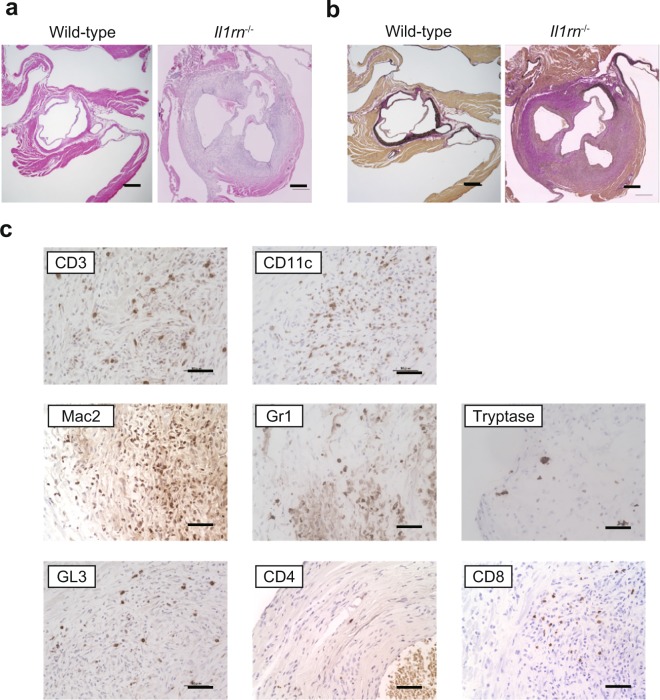
Characterization of infiltrating types of immune cells in aortitis in *Il1rn*^−/−^ mice. (**a**) H&E and (**b**) EVG staining of the aortae of wild–type and *Il1rn*^−/−^ mice (12 weeks old). Scale bars = 300 μm. (**c**) IHC using anti-CD3, CD4, CD8, CD11c, GL1, Gr1, Mac2 and tryptase Abs for local lesions of aortitis in *Il1rn*^−/−^ mice (12 weeks old). Scale bars = 20 μm. IHC, immunohistochemistry.

**Figure 2:**
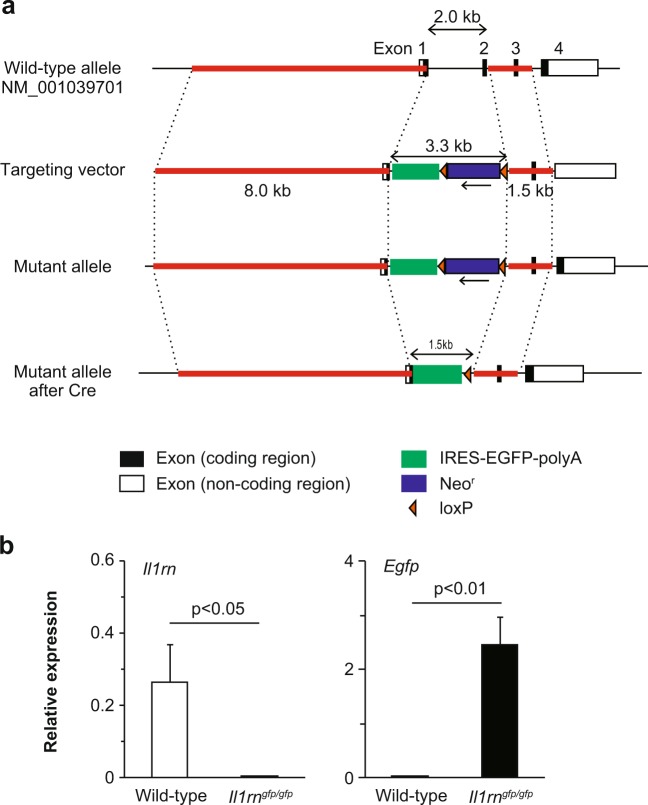
Generation of *Il1rn*^*gfp/gfp*^ mice. (**a**) IL-1Ra gene–targeting strategy. The region from a part of the sequence behind the start codon of exon 1 to a part of the intron behind exon 2 of the *II1rn* locus was replaced with a cassette consisting of IRES-EGFP and a neomycin resistance gene (*Neo*^r^), flanked by *loxP* sequences. Then the neomycin resistance gene (*Neo*^r^), flanked by *loxP* sequences, was depleted with Cre. (**b**) Expression of *Il1rn* and *Gfp* mRNA in the skin of *Il1rn*^*gfp/gfp*^ mice (n = 5, 12 weeks old), determined by qPCR. Data show the mean + SD. *p < 0.05 and **p < 0.01.

**Figure 3:**
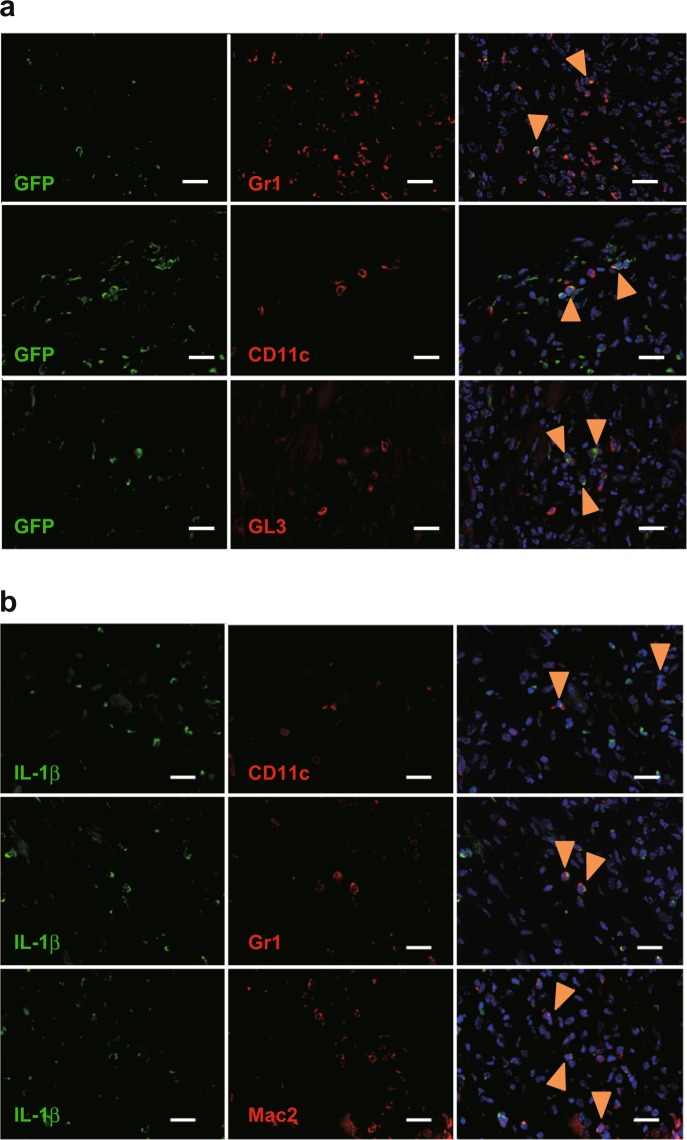
Identification of IL-1Ra– and IL-1β–producing cells in aortitis in *Il1rn*^−/−^ mice. (**a**) Expression of IL-1Ra (GFP) in Gr1^+^, CD11c^+^ and GL3^+^ cells and (**b**) expression of IL-1β in CD11c^+^, GL3^+^ and Mac2^+^ cells in local lesions of aortitis in *Il1rn*^−/−^ mice (12 weeks old). Scale bars = 20 μm. Arrowheads = Cell surface marker-positive GFP^+^ cells (**a**) and cell surface marker-positive IL-1β^+^ cells.

As reported previously^20,22,23^, *Il1rn*^−/−^ mice, but not wild–type mice or *Il25*^−/−^ mice (data not shown), developed aortitis accompanied by infiltration of immune cells into the adventitia, destruction of the elastic laminae, adventitial thickening with replacement by fibrous tissue, and neointimal thickening (Fig. 1a,b). CD3^+^ T cells, CD11c^+^ DCs, Gr1^+^ neutrophils, Mac2^+^ macrophages and tryptase^+^ mast cells had infiltrated the lesions of aortitis in *Il1rn*^−/−^ mice (Fig. 1c). In particular, CD8^+^ T cells and GL3^+^ γδT cells, but not CD4^+^ T cells, are seen (Fig. 1c). However, it remains unclear which types of cells produce IL-1Ra and IL-1β in the lesions of aortitis in *Il1rn*^−/−^ mice. For detection of IL-1Ra, we newly generated IL-1Ra–reporter mice (*Il1rn*^*gfp/gfp*^ mice; Fig. 2a), which express EGFP under the promoter of *Il1rn* genes instead of IL-1Ra (Fig. 2b) and also develop aortitis spontaneously. We found that CD11c^+^ dendritic cells (DCs), Gr1^+^ neutrophils, and GL3^+^ γδT cells—but not CD4^+^ T cells, CD8^+^ T cells or Mac2^+^ macrophages—were IL-1Ra–producing EGFP^+^ cells in the lesions of aortitis (Fig. 3a, and data not shown). On the other hand, CD11c^+^ dendritic cells (DCs), Gr1^+^ neutrophils and Mac2^+^ macrophages—but not CD4^+^ T cells, CD8^+^ T cells or GL3^+^ γδT cells—were IL-1β–producing cells (Fig. 3b, and data not shown). Neither of those cytokines was produced by non-immune cells such as endothelial cells, epithelial cells, vascular smooth muscle cells or fibroblasts (data not shown).

### Exacerbation of aortitis by IL-25

**Figure 4:**
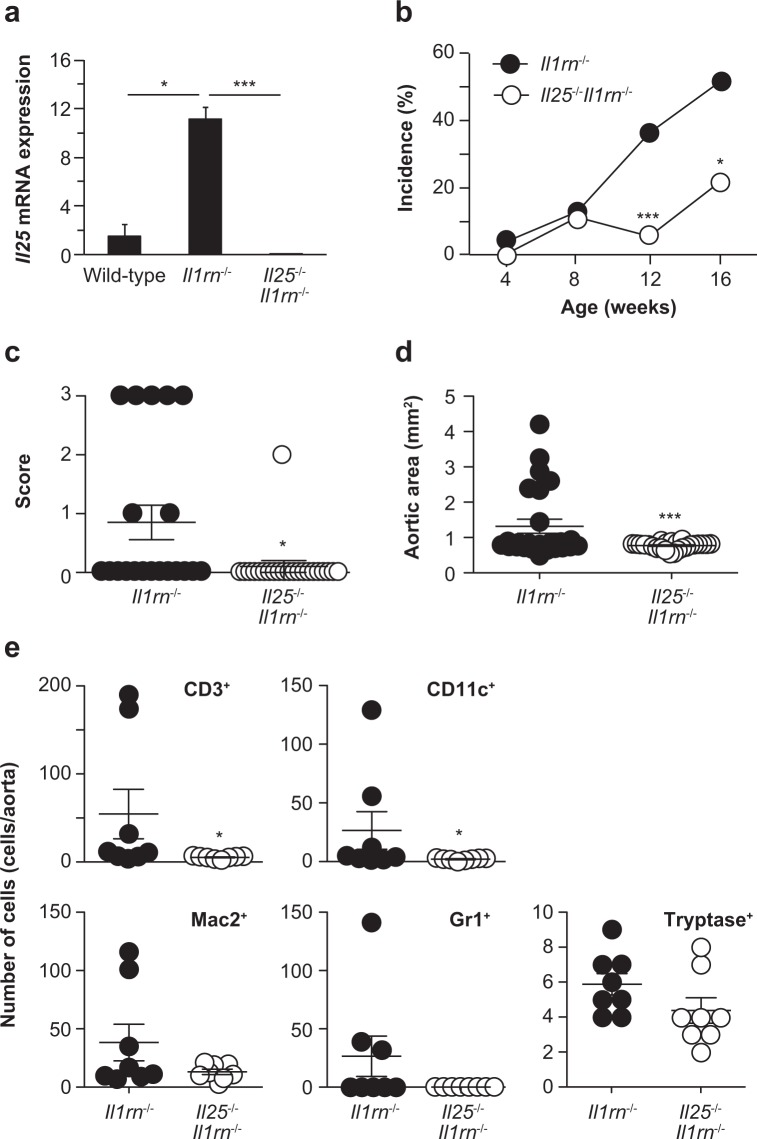
IL-25 exacerbates aortitis in *Il1rn*^−/−^ mice. (**a**) Relative expression levels of *Il25* mRNA in the aortae of wild–type (n = 8), *Il1rn*^−/−^ (n = 10) and *Il25*^−/−^*Il1rn*^−/−^ (n = 8) mice. (**b**) Incidence of aortitis. (**c**) Aortic inflamed area. (**d**) Aortitis severity score. (**e**) Numbers of CD3^+^, CD11c^+^, Mac2^+^, Gr1^+^, Mac2^+^ and tryptase^+^ cells in the aortitis of *Il1rn*^−/−^ (n = 8) and *Il25*^−/−^*Il1rn*^−/−^ (n = 8) mice (12 weeks old). Data show the mean + SD. *p < 0.05 and ***p < 0.01.

The development of aortitis in *Il1rn*^−/−^ mice is dependent mainly on IL-1 and TNF, and at least in part on IL-17A^22,23^. IL-25 can suppress or enhance certain IL-17A–mediated diseases^25,30,32,33^. We found that expression of *Il25* mRNA was significantly increased in the aortae of *Il1rn*^−/−^ mice compared with wild–type mice and *Il25*^−/−^*Il1rn*^−/−^ mice (as a negative control) (Fig. 4a), suggesting involvement of IL-25 in the development of aortitis. Indeed, the incidence of aortitis in *Il25*^−/−^*Il1rn*^−/−^ mice was significantly lower than in *Il1rn*^−/−^ mice (Fig. 4b). In association with this, the aortitis severity score and the aortic inflamed area were significantly smaller in *Il25*^−/−^*Il1rn*^−/−^ mice compared with *Il1rn*^−/−^ mice (Fig. 4c,d). In addition, the numbers of infiltrating inflammatory cells such as CD3^+^ T cells, CD11c^+^ DCs, Gr1^+^ neutrophils and Mac2^+^ macrophages—but not tryptase^+^ mast cells—in the aortae were also significantly less in *Il25*^−/−^*Il1rn*^−/−^ mice compared with *Il1rn*^−/−^ mice (Fig. 4e). These results indicate that IL-25 can exacerbate the development of aortitis.

### IL-25 enhances type 3–related cytokines, but not type 2 cytokines, in aortitis

**Figure 5:**
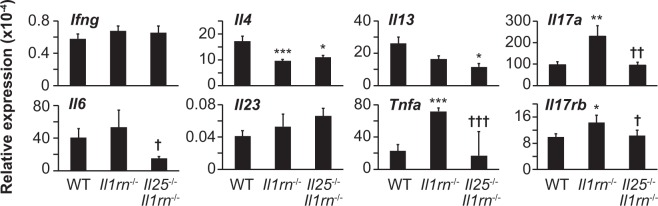
IL-25 is involved in IL-17A, IL-6 and TNF expression in aortitis in *Il1rn*^−/−^ mice. Relative mRNA expression levels of *Il17rb*, and types 1, 2, 3 and related cytokines in the aortae of wild–type (WT; n = 8), *Il1rn*^−/−^ (n = 8) and *Il25*^−/−^*Il1rn*^−/−^ (n = 8) mice (12 weeks old). Data show the mean + SD. *p < 0.05, **p < 0.01 and ***p < 0.005 vs WT mice, and ^†^ < 0.05, ^††^p < 0.01 and ^†††^p < 0.005 vs *Il1rn*^−/−^ mice.

In the aortae of *Il1rn*^−/−^ mice, the expression level of *Ifng* mRNA was comparable—but the levels of *Il4* and *Il13* mRNA were lower—compared with in wild–type mice (Fig. 5). Regarding type 3 and type 3–related cytokines such as IL-17A, IL-6, IL-23p19 and TNF, the expression levels of *Il17a* and *Tnfa* mRNA were significantly higher, while those of *Il6* and *Il23p19* mRNA were comparable, in the aortae of *Il1rn*^−/−^ mice compared with wild–type mice (Fig. 4a). Although IL-25 is known to induce production of type 2 cytokines by various types of cells^27,28^, the expression levels of *Il4* and *Il13* mRNA were comparable in the aortae of *Il25*^−/−^*Il1rn*^−/−^ mice and *Il1rn*^−/−^ mice (Fig. 5), suggesting that IL-25 is not crucial for induction of such type 2 cytokines in the lesions of aortitis. In addition, the expression levels of *Ifng*, *Il6* and *Il23p19* mRNA were comparable in the aortae of *Il1rn*^−/−^ mice and *Il25*^−/−^*Il1rn*^−/−^ mice (Fig. 5). On the other hand, the expression levels of *Il6*, *Il17a* and *Tnfa* mRNA were significantly reduced in *Il25*^−/−^*Il1rn*^−/−^ mice compared with *Il1rn*^−/−^ mice (Fig. 5). IL-25 was reported to inhibit IL-13–dependent Th17 cell differentiation, thereby contributing to suppression of Th17–mediated autoimmune diseases^25,30^. On the other hand, our results suggest that IL-25 may enhance production of IL-6, IL-17A and TNF, but not IL-23, thereby contributing to development of IL-1–, TNF– and IL-17A–dependent aortitis in *Il1rn*^−/−^ mice.

### Identification of IL-25R-expressing cells in lesions of aortitis

**Figure 6:**
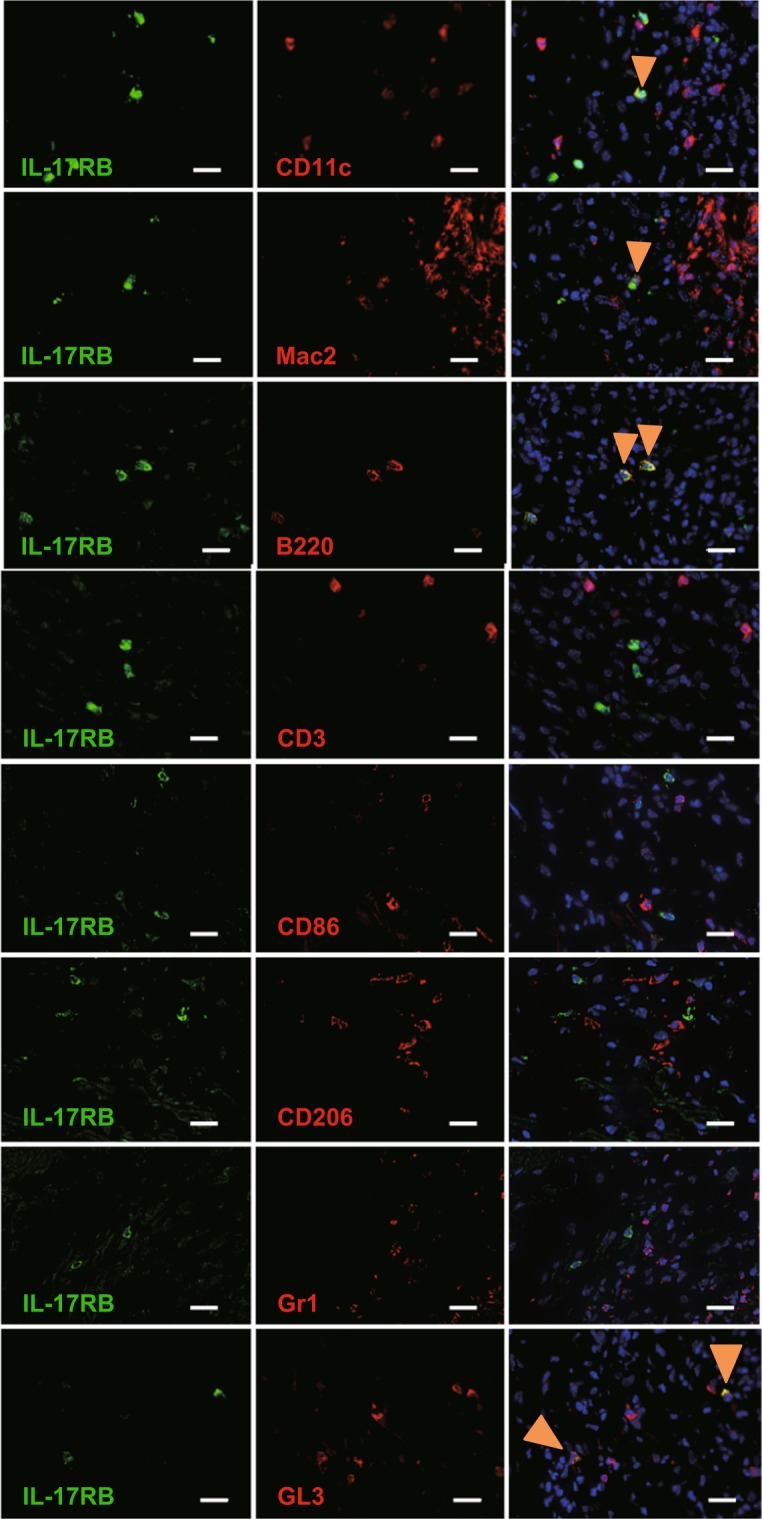
Identification of IL-25 receptor–expressing cells in aortitis in *Il1rn*^−/−^ mice. IL-17RB was expressed in CD11c^+^, Mac2^+^, B220^+^ and GL3^+^ cells—but not CD3^+^, CD86^+^, CD206^+^ or Gr1^+^cells—in a local lesion of aortitis in *Il1rn*^−/−^ mice (12 weeks old). Scale bars = 20 μm. Arrowheads = Cell surface marker-positive IL-17RB^+^ cells

mRNA expression of IL-17RB, which is a component of IL-25R, was increased in the aortae of *Il1rn*^−/−^ mice compared with wild–type mice (Fig. 5). In addition, IL-17RB was expressed on some, but not all, CD11c^+^ DCs, Mac2^+^ macrophages, B220^+^ B cells and GL3^+^ γδT cells in the aortae of *Il1rn*^−/−^ mice (Fig. 6), while it was not detected on CD3^+^ T cells or Gr1^+^ neutrophils (Fig. 6). Among macrophages, IL-17RB was not expressed on CD206^+^ M2 macrophages or CD86^+^ M1 macrophages in the aortae of *Il1rn*^−/−^ mice (Fig. 6).

### Importance of non-immune cell-derived IL-25 for development of aortitis

**Figure 7:**
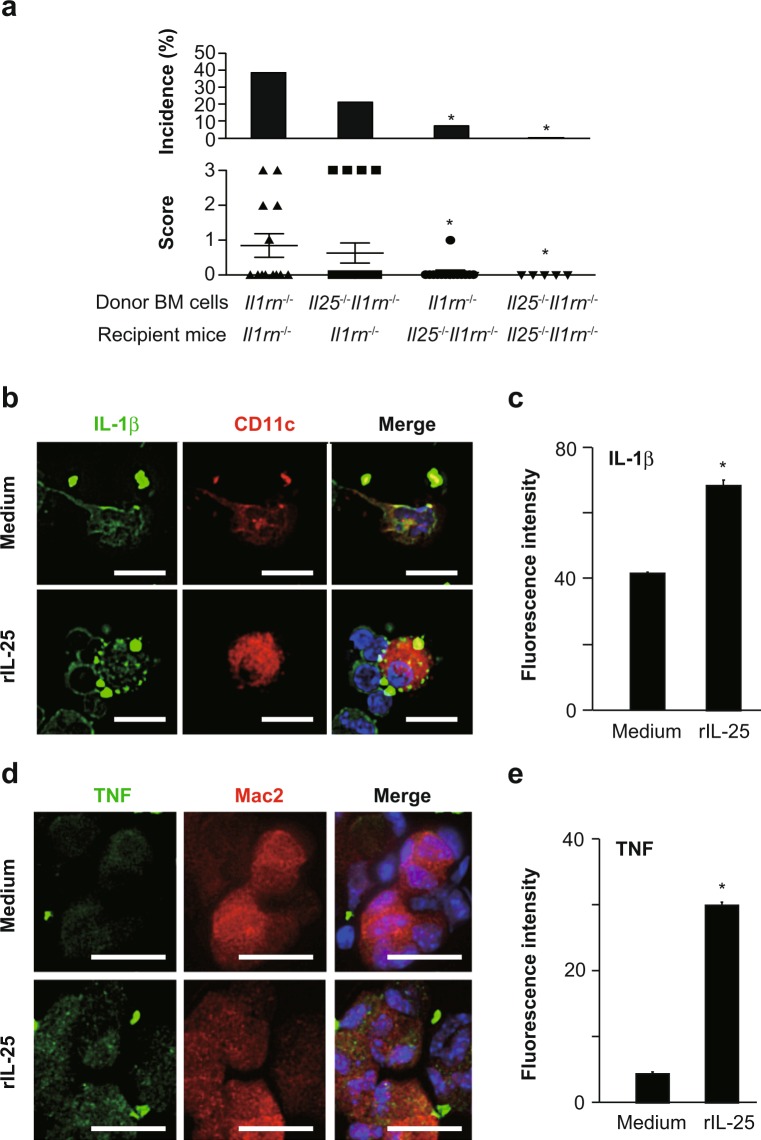
IL-25 responder and producer cells in aortitis in *Il1rn*^−/−^ mice. (**a**) Incidence and severity score of aortitis in *Il1rn*^−/−^ mice transferred with *Il1rn*^−/−^ BM cells (n = 13) or *Il25*^−/−^*Il1rn*^−/−^ BM cells (n = 19) and in *Il25*^−/−^*Il1rn*^−/−^ mice transferred with *Il1rn*^−/−^ BM cells (n = 14) or *Il25*^−/−^*Il1rn*^−/−^ BM cells (n = 5). Data show the mean + SD. *p < 0.05 vs. *Il1rn*^−/−^ mice transferred with *Il1rn*^−/−^ BM cells. (**b,c**) Expression of IL-1β in CD11c^+^ DCs and (**d,e**) expression of TNF in Mac2^+^ macrophages prepared from the aortae of *Il1rn*^−/−^ mice after IL-25 stimulation. Data show the mean + SD (n = 130 fields; c and e). *p < 0.001 vs medium alone. Scale bars = 20 μm.

Although *Il25* mRNA expression was significantly increased in the aortae of *Il1rn*^−/−^ mice (Fig. 3a), IL-25 proteins were below the limit of detection by immunohistochemical analysis of those aortic specimens (data not shown). To identify the cells producing IL-25 in the aortae of *Il1rn*^−/−^ mice, we performed bone marrow (BM) cell transfer analysis. *Il1rn*^−/−^ mice transferred with *Il25*^−/−^*Il1rn*^−/−^ BM cells developed aortitis similarly to *Il1rn*^−/−^ mice transferred with *Il1rn*^−/−^ BM cells (Fig. 7a), indicating that IL-25 produced by BM cell–derived immune cells was not essential for development of aortitis in *Il1rn*^−/−^ mice. On the other hand, like *Il25*^−/−^*Il1rn*^−/−^ mice transferred with *Il25*^−/−^*Il1rn*^−/−^ BM cells, *Il25*^−/−^*Il1rn*^−/−^ mice transferred with *Il1rn*^−/−^ BM cells showed reduced development of aortitis compared with *Il1rn*^−/−^ mice transferred with *Il1rn*^−/−^ BM cells (Fig. 7a), indicating that IL-25 produced by non-immune cells is crucial for the setting.

### IL-25 enhances production of IL-1β by dendritic cells and TNF by macrophages

We next investigated the roles of IL-25 in production of IL-1β by DCs, which were shown to produce IL-1β and to express IL-17RB in the aortae of *Il1rn*^−/−^ mice (Figs. 3b and 6), and in production of TNF by M2 macrophages, which express IL-17RB and produce TNF in response to IL-25 in humans^34^. Therefore, we isolated single cells from the aortae of *Il1rn*^−/−^ mice and cultured them in the presence and absence of IL-25. Immunohistochemical analysis showed increased expression of IL-1β in CD11c^+^ DCs and of TNF in Mac2^+^ macrophages derived from the aortae of *Il1rn*^−/−^ mice after IL-25 stimulation (Fig. 7b–e). These observations suggest that non-immune cell–derived IL-25 is crucial for production of IL-1β by IL-17RB^+^ CD11c^+^ DCs and production of TNF by IL-17RB^+^ Mac2^+^ macrophages, and that it contributes to development of aortitis in *Il1rn*^−/−^ mice.

## Discussion

*Il1rn*^−/−^ mice spontaneously develop aortitis resembling such LVV as TAK and GCA; that is, infiltration of inflammatory cells into all aortic layers, necrosis of vascular smooth muscle cells, disruption of elastic fibers, and vascular remodeling, including stenosis of the vascular lumen and aortic dilatation^1^ (Fig. 1a). It was reported that the development of aortitis was diminished in *Il1r1*^−/−^ *Il1rn*^−/−^ mice and *Tnfa*^−/−^ *Il1rn*^−/−^ mice, whereas it was normal in *Il6*^−/−^ *Il1rn*^−/−^ mice^21,22^, indicating that IL-1α and/or IL-1β, and TNF are crucial for development of aortitis in *Il1rn*^−/−^ mice. T cells and IL-17A are also known to be important for development of aortitis in *Il1rn*^−/−^ mice^22,23^. Since IL-1 and/or TNF can activate Th17 cells^35^, it is thought that IL-1– and/or TNF–induced T cell–derived IL-17 may be involved in development of aortitis in *Il1rn*^−/−^ mice. In the present study, we demonstrated that IL-25 is crucial for development of IL-1–, TNF– and IL-17–mediated aortitis in *Il1rn*^−/−^ mice.

We found that expression of *Il25* mRNA was significantly increased in the aortae of *Il1rn*^−/−^ mice compared with wild–type mice (Fig. 4a). It has been reported that the source of IL-25 is resident microglia and/or brain capillary endothelial cells in EAE and/or MS^30,36^. In addition, IL-25 was expressed in lung and/or nasal epithelial cells of mice and/or humans^37–39^, in B cells, smooth muscle cells and endothelial cells in atherosclerotic arteries of humans^40^, in keratinocytes of patients with atopic dermatitis^41^, and in subepithelial macrophage-like cells and epithelial cells in the human colon^42^. We previously reported that IL-25 is crucial for development of allergic contact dermatitis as well as asthma in mice^32,38^. Although IL-25 proteins were detectable in the lung during asthma^38^, they were hardly detectable in the skin during allergic contact dermatitis^32^. Like in allergic contact dermatitis, IL-25 proteins were hardly detectable in aortae of *Il1rn*^−/−^ mice (12 weeks) by immunofluorescence using anti-IL-25 Ab (data not shown). In addition to such non-immune cells as epithelial cells, endothelial cells, fibroblasts and tuft cells, immune cells such as mast cells, eosinophils and alveolar macrophages (M2) are known to be sources of IL-25^43–45^. Although there was macrophage infiltration in the aortitis of *Il1rn*^−/−^ mice (Fig. 1c), CD206^+^ M2 macrophages were not observed among them (Fig. 6). Infiltrating granulocytes in the aortitis of *Il1rn*^−/−^ mice were overwhelmingly neutrophils (Fig. 1c), not eosinophils (data not shown). Mast cells were observed in the aortitis of *Il1rn*^−/−^ mice, but their number was relatively small compared with macrophages and neutrophils (Figs. 1c and 4e). Regarding this, using bone marrow cell transfer analysis, we showed that IL-25 derived from non-hematopoietic cells rather than immune cells was crucial for development of IL-1–, TNF– and IL-17–mediated aortitis in *Il1rn*^−/−^ mice. Since the levels of IL-25 proteins in bronchoalveolar lavages from mice with ILC2–dependent innate–type airway inflammation and the levels of *Il25* mRNA in the skin of mice with imiquimod-induced psoriatic dermatitis peaked at one hour after antigen challenge^46,47^, IL-25 production in the onset phase of aortitis in *Il1rn*^−/−^ mice (i.e., 4 weeks) may be important for development of the disease.

IL-25 can induce such type 2 cytokines as IL-4, IL-5 and IL-13 by various types of cells, including Th2 cells^27,28^. It is well known that Th2 cells suppress Th1 cell– and Th17 cell–mediated immune responses. In addition, IL-25 can inhibit IL-13–dependent Th17 cell differentiation, contributing to suppression of Th17–mediated autoimmune diseases^25,30,31^. Since accumulation of Th2 cells as well as Th1 cells was observed in inflamed lesions of aortitis in *Il1rn*^−/−^ mice^21^, we suspected that IL-25 may be involved in suppression of IL-1–, TNF– and IL-17–mediated aortitis in *Il1rn*^−/−^ mice by enhancing Th2 cell activation. However, *Il25*^−/−^ *Il1rn*^−/−^ mice showed attenuated development of aortitis compared with *Il1rn*^−/−^ mice (Fig. 4b), indicating that IL-25 contributed to exacerbation—not suppression—of IL-1–, TNF– and IL-17–mediated aortitis in *Il1rn*^−/−^ mice. Intraperitoneal IL-25 administration to mice resulted in induction of such type 2 cytokines as IL-4, IL-5 and/or IL-13 in the spleen, stomach, small intestine, kidney and liver^48^. On the other hand, IL-25 injection into the skin of mice resulted in induction of type 3–related cytokines such as IL-1β, IL-6, and TNF and neutrophil chemoattractant factors^32,47^. In our model, IL-25 deficiency resulted in reduced expression of *Il17a* and *Tnfa* mRNA in the aortae of *Il1rn*^−/−^ mice, suggesting that IL-25 somehow enhanced the development of IL-1–, TNF– and IL-17–mediated aortitis in *Il1rn*^−/−^ mice.

As shown in Fig. 6, we demonstrated that type 2 immune cells (CD3^+^ T cells, including Th2 cells, and CD206^+^ M2-macrophages) did not express IL-25 receptor (IL-17RB). Thus, it seemed that IL-25 cannot activate such immune cells to induce type 2 immune responses in the aortae of *Il1rn*^−/−^ mice. On the other hand, in the local lesions of aortitis in *Il1rn*^−/−^ mice, we identified CD11c^+^ DCs, Mac2^+^ macrophages, γδ T cells and B cells as IL-25 receptor (IL-17RB)–expressing cells (Fig. 6). We cultured GM-CSF–induced and GM-CSF plus IL-4–induced BM cell–derived DCs (BMDCs) and TNF– and LPS–treated BMDCs in the presence of IL-25 *in vitro*. However, we found that IL-25 did not induce production of IL-1β or TNF by those cells (data not shown), because *Il17rb* mRNA was hardly detected by qPCR (data not shown). In addition, IL-17RB was expressed on M2—but not M0 or M1—macrophages derived from human PBMCs, and the M2 macrophages produced TNF, IL-6 and certain chemokines in response to IL-25^34^. We generated M2 and M0 macrophages from BM cells in the presence of rmM-CSF and rmIL-4, respectively, and then stimulated them *in vitro* with IL-25. However, we found that IL-25 did not induce TNF production by either of those macrophages because their expression of *Il17rb* mRNA was below the limit of detection by qPCR (data not shown). Consistent with this, IL-17RB was not expressed on CD206^+^ M2 macrophages or CD86^+^ M1 macrophages from the aortae of *Il1rn*^−/−^ mice (Fig. 6). Thus, IL-17RB^+^ Mac2^+^ macrophages seen in the aortae of *Il1rn*^−/−^ mice were a distinct population from the CD206^+^ M2 and CD86^+^ M1 macrophages. Likewise, γδ T cells purified from the peritoneal cavity of naïve wild–type mice did not produce IL-17A in response to IL-25, since expression of *Il17rb* mRNA was below the limit of detection by qPCR (data not shown). Further study is needed to elucidate the contribution of IL-17RB^+^ γδ T cells in local lesions to development of aortitis in *Il1rn*^−/−^ mice. Since no CD3^+^ T cells, including Th2 cells, in the aortae expressed IL-25R, Th2 cells apparently do not produce IL-13 in the aortae of *Il1rn*^−/−^ mice. On the other hand, IL-25R-expressing DCs and macrophages can produce IL-1β and TNF, respectively, in response to IL-25 (Fig. 7). DC-derived IL-1β and macrophage-derived TNF are thought to subsequently activate Th17 cells to induce IL-17, contributing to development of aortitis in *Il1rn*^−/−^ mice. Therefore, the function of IL-25 is reflected by the types of IL-25R-expressing cells in the local site.

As shown in Fig. 4b, the development of aortitis was attenuated, but not completely abrogated, in *Il25*^−/−^ *Il1rn*^−/−^ mice compared with *Il1rn*^−/−^ mice. Recently, it was reported that IL-17B, which (like IL-25) is a ligand for IL-17RB, is crucial for development of bleomycin-induced pulmonary fibrosis in mice by promoting type 3 immune responses in mice^49^. Therefore, IL-17B may have a compensatory role in induction of type 3 immune responses in the aortitis of *Il25*^−/−^ *Il1rn*^−/−^ mice.

In the present study, we identified the steps of a new function of IL-25: 1. Non-immune cell–derived IL-25 can induce production of IL-1β and TNF by IL-17RB–expressing DCs and macrophages. 2. DC-derived IL-1β and macrophage-derived TNF can activate Th17 cells to produce IL-17A. And 3. Th17 cell-derived IL-17A contributes to development of aortitis in *Il1rn*^−/−^ mice. Our findings improve our understanding of the molecular mechanisms involved in development of aortitis and suggest that neutralization of IL-25 may be a potential therapeutic target for aortitis.

## Methods

### Mice

BALB/cNcr–wild-type mice were purchased from Japan SLC, Inc. (Shizuoka, Japan). *Il1rn*^−/−^ mice and *Il25*^−/−^ mice on the BALB/c background were generated as described previously^50,51^, and they were crossed to generate *Il25*^−/−^*Il1rn*^−/−^ mice. All mice were housed under specific–pathogen–free conditions in an environmentally–controlled animal room and fed a normal chow diet at the Institute of Medical Science, The University of Tokyo. The animal protocols for experiments were approved by the Institutional Review Board of the Institute (A16–23 and A16–30), and all experiments were conducted according to the ethical and safety guidelines of the Institute.

### Generation of *Il1rn*^*gfp/gfp*^ mice

The *Il1rn* gene (NM_001039701) was disrupted by replacement of the region from a part of the sequence behind the start codon of exon 1 to a part of the intron behind exon 2 with a cassette consisting of IRES-EGFP and a neomycin resistance gene, flanked by *loxP* sequences. Homologous regions were amplified by PCR using the following primers: 5′-CTGAAAGAAGGAATCAGAAACAGC-3′ and 5′- GGGTCTTTTCCCAGAAGGGCGGCAGG-3′ to generate an 8-kb fragment, and 5′-GACATAGAGTCCTTTGCCCTGCTC-3′, and 5′-GAAGGTAGGCTCAACTGGTTTAGG-3′ to generate a 1.5-kb fragment. The targeting vector was electroporated into BALB/c ES cells (SCC052, Merck). Male chimeric mice were obtained from two distinct targeted clones and mated with BALB/cNcr female mice. Genotyping of *Il1rn*^−/−^ mice was performed by PCR using the following primers: common (5′-AATGAGGACATCCCACCTCCAGGC-3′), WT (5′-ACTATAGGATGTGCTTGCATCGCC-3′), and MT (5′-GACGTGCTACTTCCATTTGTCACG-3′). The common and WT primers were used for detection of wild–type alleles (479 bp), and the common and MT primers were used for detection of mutant alleles (383 bp). Then a plasmid carrying Cre cDNA (pCAG-Cre; kindly provided by Dr. Jun-ichi Miyazaki, Osaka University) was injected into fertilized eggs from the *Il1rn*^−/−^ mice to deplete the neomycin resistance gene, and it was flanked by *loxP* sequences. The eggs were then transferred into pseudopregnant female BALB/c mice. Genotyping of *Il1rn*^*gfp/gfp*^ mice was performed by PCR using the following primers: common 2 (5′-GGAACGGAATGACAGCAGCACAGG-3′), WT2 (5′-TATATCTCCTATTCCTGCATATGCTC-3′), and MT2 (5′-CGACACCGGCCTTATTCCAAGCGG-3′). The common 2 and WT2 primers were used for detection of wild-type alleles (325 bp), and the common 2 and MT2 primers were used for detection of mutant alleles (269 bp).

### Scoring of aortitis

Mice were anesthetized with sevoflurane (Pfizer, Japan) and then perfused with phosphate–buffered saline (PBS). The aortae and hearts were then embedded in Optimal Cutting Temperature (OCT) compound (Tissue-Tek; Sakura Finetek Japan Co., Tokyo, Japan) and frozen in liquid nitrogen and an isopentane slurry. Then frozen coronal sections (7-μm thickness) were prepared with Cryostat (Leica) and stored at −80 °C until use. The sections were stained with hematoxylin and eosin (H&E) according to the standard protocol and with Elastica van Gieson (EVG) according to the manufacturer’s recommendations (Muto Pure Chemicals, Japan). The aortic inflamed area was evaluated using a BZ-X710 microscope and software (KEYENCE, Japan). The severity of aortitis was scored as follows: 0, no inflammation; 1, rare inflammatory cells infiltrating the intimal layer or tunica adventitia layer; 2, infiltration into the tunica media layer; and 3, diffuse infiltration by inflammatory cells.

For immunohistochemistry (IHC), the frozen coronal sections were fixed in 4% paraformaldehyde (PFA) at room temperature (RT) for 10 minutes. The sections were blocked with Blocking One Histo (Nacalai, Japan) to prevent non-specific Ab binding at RT for 10 min, followed by incubation with primary Abs: rabbit anti-mouse CD3 pAb (ab5690; Abcam plc.); rabbit anti-mouse CD4 mAb (25229; Cell Signaling Technology); rabbit anti-CD8 mAb (98941; Cell Signaling Technology); rabbit anti-mouse CD11c pAb (97585; Cell Signaling Technology); rat anti-mouse Ly-6G/-6C mAb (ab2557; Abcam plc.); rat anti-mouse Mac-2 (CL8942AP; Cedarlane); hamster anti-mouse GL3 mAb (118101; Biolegend); rabbit anti-tryptase mAb (ab134932; Abcam plc.); rabbit anti-mannose pAb (ab64693; Abcam plc); rat anti-CD86 mAb (ab119857; Abcam plc); and rat anti-CD45R mAb (550286; BD Pharmingen). The sections were then incubated at 4 °C overnight in a PBS–humidified staining box. The sections were next blocked with a 0.3% hydrogen peroxide solution and 0.1% sodium azide at RT for 10 minutes to inhibit endogenous peroxidase activities. After washing, the specimens were incubated at RT for 30 minutes with secondary Abs: N-Histofine Simple Stain MAX PO Rat (414311; Nichirei Biosciences Inc.); Dako EnVision + System-HRP Labeled Polymer Anti-rabbit (K4002; Dako); N-Histofine Simple Stain MAX PO Goat (414351; Nichirei Biosciences Inc.); and Polink-2 Plus HRP Armenian Hamster with DAB kit (D87–6; Golden Bridge International). The specimens were developed using ImmPACT™ DAB (SK-4105; Vector Laboratories, Burlingame, CA). The sections were counterstained with Mayer’s hematoxylin at RT for 1 minute. Samples were mounted on slides using Malinol (20092; Muto Pure Chemicals, Japan) and imaged using a BZ-X710 (KEYENCE, Japan). Cells (Ab-positive cells per section) in the cross-section of the aortae were counted.

For immunofluorescence (IF) of aortae, the frozen coronal sections were fixed in 4% PFA at RT for 10 minutes. The sections were blocked at RT for 10 min with Blocking One Histo (Nacalai, Japan) to prevent non-specific Ab binding, followed by incubation overnight at 4 °C with primary Ab (the Abs for cell surface markers described above), chicken anti-GFP pAb (ab13970; Abcam plc.), biotin-conjugated anti-mouse IL-25 pAb (BAF1399; R&D Systems), biotin-conjugated anti-mouse IL-17RB pAb (BAF1040; R&D Systems), and goat anti-IL-1β pAb (AF401; R&D Systems). After washing, the samples were incubated with secondary Abs at RT for 45 minutes: Alexa Fluor® 488-conjugated streptavidin (S11223; Thermo Fisher Scientific); Alexa Fluor® 488-conjugated donkey anti-rat (ab150153; Abcam plc.); Alexa Fluor® 594-conjugated donkey anti-rabbit (ab150064; Abcam plc.); Alexa Fluor® 594-conjugated donkey anti-rat (ab150156; Abcam plc.); Alexa Fluor® 594-conjugated goat anti-hamster (A21113; Thermo Fisher Scientific); Alexa Fluor® 488-conjugated goat anti-chicken (ab150169, Abcam plc); and Alexa Fluor® 647-conjugated donkey anti-goat (ab150135; Abcam plc). For biotinylated antibodies, endogenous biotin was blocked according to the manufacturer’s protocol (SP-2001; Vector Laboratories, Inc.). The cell nuclei were counter-stained using NucBlue Fixed Cell Stain ReadyProbes reagent (1890342; Thermo Fisher Scientific) at RT for 30 minutes. Then the samples were mounted on slides using Dako Fluorescence Mounting Medium (S3023; Dako, Japan) and analyzed with a BZ-X710 microscope (4×, 20×, 40×, 80× original magnification; KEYENCE, Japan) and software (BZ-X800 analyzer; KEYENCE, Japan).

For IF of aortic cells, *Il1rn*^−/−^ mice were deeply anesthetized with isoflurane and 100% oxygen and perfused intracardially with ice-cold PBS to remove blood cells. Then the aortae (pooled from 10 mice) were dissected and enzymatically digested in HBSS containing 1 mg/ml type II collagenase (Worthington Biochemical Corporation, NJ), 4.5 units of elastase (Worthington Biochemical Corporation) and 30 μg/ml DNase I (Sigma-Aldrich) at 37 °C for 1 hour on a shaker at 60 rpm. The cell suspensions of the digested aortae were then passed through a 40-μm–pore cell strainer (Greiner Bio-One). The cells were cultured in RPMI1640 medium containing 10% FBS and penicillin/streptomycin in the presence and absence of 100 ng/ml rmIL-25 (R&D Systems) at 37 °C for 6 hours. After cultivation, the cells were collected and adhered to microscope slides by centrifugation with Cytospin (Thermo Scientific) at 400 rpm for 5 min. The specimens were fixed in Phosflow (BD Bioscience) at RT for 15 min and blocked with Blocking One Histo (Nacalai) at RT for 10 min. After washing, the specimens were permeabilized with 0.1% Tween 20 at RT for 5 min, followed by incubation with primary Abs (rabbit anti-mouse CD11c pAb, and goat anti-IL-1β pAb) and secondary Abs (Alexa Fluor® 488-conjugated donkey anti-goat Ig, and Alexa Fluor® 594-conjugated donkey anti-rabbit Ig), as described above. The cell nuclei were counter-stained using NucBlue Fixed Cell Stain ReadyProbes reagent (Thermo Fisher Scientific) at RT for 30 minutes. Then the samples were mounted on slides using Dako Fluorescence Mounting Medium (Dako, Japan) and analyzed with a BZ-X710 microscope, as described above.

### BM cell transplantation

BM cells were collected from the femurs, tibias and pelvises of donor mice (5 to 6 weeks old). Recipient mice (4 weeks old) were lethally irradiated with one dose of 7.5 Gy X-rays and then injected retro-orbitally with the BM cells (2.0 × 10^7^ cells/mouse) in 0.15 ml of PBS under anesthesia by continuous inhalation of isoflurane (Pfizer, Japan).

### Quantitative polymerase chain reaction (qPCR)

NucleoSpin RNA Plus XS (Takara, Japan) was used to prepare total RNA from the residual frozen aortae that had been embedded in OCT compound for histological analysis, as described above. cDNA was transcribed from purified RNA using a ReverTra Ace qPCR RT Master Mix (TOYOBO, Japan). qPCR was performed using SYBR Premix Dimer Eraser (Takara) and a CFX 384 Touch Real-time PCR Detection System (Bio-Rad, Hercules, CA) according to the manufacturers’ instructions. For detection of *Il25* mRNA, qPCR analysis was performed twice, as follows. After the first round of qPCR, the PCR products were purified by NucleoSpin Gel and PCR Clean-up (Takara). Then a second round of qPCR was performed using the purified PCR products. Amplification reactions were performed in duplicate, and all mRNA expression levels were calculated using the comparative CT method formula 2^-ΔΔct^. The data were normalized to the level of *Gapdh* mRNA. The designed primers are shown in Table 1.

**Table 1:** Sequences of primers.

Gene	Forward (5′-3′)	Reverse (5′-3′)
*Ifng*	GAACTGGCAAAAGGATGGTGA	TGTGGGTTGTTGACCTCAAAC
*Il1b*	CAACCAACAAGTGATATTCTCCATG	GATCCACACTCTCCAGCTGCA
*Il1rn*	GCTCATTGCTGGGTACTTACAA	CCAGACTTGGCACAAGACAGG
*Il4*	TCCAAGGTGCTTCGCATATTTT	CAGCTTATCGATGAATCCAGGC
*Il6*	GAGGATACCACTCCCAACAGACC	AAGTGCATCATCGTTGTTCATACA
*Il13*	GGCAGCAGCTTGAGCACATT	GGCATAGGCAGCAAACCATG
*Il17a*	CCGCAATGAAGACCCTGATAGAT	AGAATTCATGTGGTGGTCCAGC
*Il17rb*	GGCTGCCTAAACCACGTAATG	CCCGTTGAATGAGAATCGTGT
*Il23*	GGTGGCATCGAGAAACTGTGA	GCTGTCTGGAGTACTGTGCATCTG
*Il25*	AAGTGGAGCTCTGCATCTGT	CGATTCAAGTCCCTGTCCAA
*Tnfa*	GCCTCCCTCTCATCAGTTCT	CACTTGGTGGTTTGCTACGA
*Gapdh*	CCCACTCTTCCACCTTCGATG	AGGTCCACCACCCTGTTGCT
*Egfp*	CAGCTCGCCGACCACTACC	TTACTTGTACAGCTCGTCCATG

### Statistical analysis

All values were calculated as the mean ± SD except where indicated otherwise. Fisher’s exact test was used for evaluation of the incidence of aortitis between unpaired groups. Unless otherwise specified, the unpaired Student’s t test, two-tailed, were used.

## References

[1] Stone JR (2015). Consensus statement on surgical pathology of the aorta from the Society for Cardiovascular Pathology and the Association for European Cardiovascular Pathology: I. Inflammatory diseases. Cardiovascular pathology: the official journal of the Society for Cardiovascular Pathology.

[2] Miller DV, Maleszewski JJ (2011). The pathology of large-vessel vasculitides. Clinical and experimental rheumatology.

[3] de Boysson H (2018). Large-vessel involvement and aortic dilation in giant-cell arteritis. A multicenter study of 549 patients. Autoimmun Rev.

[4] Mazlumzadeh M (2006). Treatment of giant cell arteritis using induction therapy with high-dose glucocorticoids: a double-blind, placebo-controlled, randomized prospective clinical trial. Arthritis Rheum.

[5] Mukhtyar C (2009). EULAR recommendations for the management of large vessel vasculitis. Ann Rheum Dis.

[6] Hoffman GS (1994). Treatment of glucocorticoid-resistant or relapsing Takayasu arteritis with methotrexate. Arthritis Rheum.

[7] Mahr AD (2007). Adjunctive methotrexate for treatment of giant cell arteritis: an individual patient data meta-analysis. Arthritis Rheum.

[8] Ohigashi H (2017). Effects of immunosuppressive and biological agents on refractory Takayasu arteritis patients unresponsive to glucocorticoid treatment. J Cardiol.

[9] Samson M (2018). Biological treatments in giant cell arteritis & Takayasu arteritis. Eur J Intern Med.

[10] Comarmond C (2012). Anti TNF-alpha in refractory Takayasu’s arteritis: cases series and review of the literature. Autoimmun Rev.

[11] Clifford A, Hoffman GS (2014). Recent advances in the medical management of Takayasu arteritis: an update on use of biologic therapies. Current opinion in rheumatology.

[12] Hoffman GS (2007). Infliximab for maintenance of glucocorticosteroid-induced remission of giant cell arteritis: a randomized trial. Ann Intern Med.

[13] Martinez-Taboada VM (2008). A double-blind placebo controlled trial of etanercept in patients with giant cell arteritis and corticosteroid side effects. Ann Rheum Dis.

[14] Seror R (2014). Adalimumab for steroid sparing in patients with giant-cell arteritis: results of a multicentre randomised controlled trial. Ann Rheum Dis.

[15] Noris M (2001). Pathogenesis of Takayasu’s arteritis. Journal of nephrology.

[16] Blain H (2002). Arterial wall production of cytokines in giant cell arteritis: results of a pilot study using human temporal artery cultures. J Gerontol A Biol Sci Med Sci.

[17] Hernandez-Rodriguez J (2004). Tissue production of pro-inflammatory cytokines (IL-1beta, TNFalpha and IL-6) correlates with the intensity of the systemic inflammatory response and with corticosteroid requirements in giant-cell arteritis. Rheumatology.

[18] Soto Lopez ME (2013). The interleukin-1 gene cluster polymorphisms are associated with Takayasu’s arteritis in Mexican patients. J Interferon Cytokine Res.

[19] Alvarez-Rodriguez L (2009). Interleukin-1RN gene polymorphisms in elderly patients with rheumatic inflammatory chronic conditions: Association of IL-1RN*2/2 genotype with polymyalgia rheumatica. Hum Immunol.

[20] Nicklin MJ, Hughes DE, Barton JL, Ure JM, Duff GW (2000). Arterial inflammation in mice lacking the interleukin 1 receptor antagonist gene. J Exp Med.

[21] Shepherd J, Nicklin MJ (2005). Elastic-vessel arteritis in interleukin-1 receptor antagonist-deficient mice involves effector Th1 cells and requires interleukin-1 receptor. Circulation.

[22] Matsuki T (2005). Involvement of tumor necrosis factor-alpha in the development of T cell-dependent aortitis in interleukin-1 receptor antagonist-deficient mice. Circulation.

[23] Ishigame, H. *et al*. The role of TNFalpha and IL-17 in the development of excess IL-1 signaling-induced inflammatory diseases in IL-1 receptor antagonist-deficient mice. *Ernst Schering Research Foundation workshop*, 129–153 (2006). 10.1007/3-540-37673-9_8 16329650

[24] Ly KH (2014). Interleukin-1 blockade in refractory giant cell arteritis. Joint Bone Spine.

[25] Zaph C (2008). Commensal-dependent expression of IL-25 regulates the IL-23-IL-17 axis in the intestine. J Exp Med.

[26] Paul WE, Zhu J (2010). How are T(H)2-type immune responses initiated and amplified?. Nature reviews. Immunology.

[27] Reynolds, J. M., Angkasekwinai, P. & Dong, C. IL-17 family member cytokines: regulation and function in innate immunity. *Cytokine Growth Factor Rev*, **21**, 413–423, doi:S1359-6101(10)00069-9 [pii] 10.1016/j.cytogfr.2010.10.002 [doi] (2010). 10.1016/j.cytogfr.2010.10.002 PMC3008409 21074482

[28] Klose CS, Artis D (2016). Innate lymphoid cells as regulators of immunity, inflammation and tissue homeostasis. Nat Immunol.

[29] Saenz SA, Taylor BC, Artis D (2008). Welcome to the neighborhood: epithelial cell-derived cytokines license innate and adaptive immune responses at mucosal sites. Immunol Rev.

[30] Kleinschek, M. A. *et al*. IL-25 regulates Th17 function in autoimmune inflammation. *J Exp Med*, **204**, 161–170, doi:jem.20061738 [pii] 10.1084/jem.20061738 [doi] (2007). 10.1084/jem.20061738 PMC2118427 17200411

[31] Liu D (2016). IL-25 attenuates rheumatoid arthritis through suppression of Th17 immune responses in an IL-13-dependent manner. Sci Rep.

[32] Suto, H. *et al*. IL-25 enhances TH17 cell-mediated contact dermatitis by promoting IL-1beta production by dermal dendritic cells. *J Allergy Clin Immunol*, **142**, 1500–1509 e1510, doi:S0091-6749(18)30326-9 [pii] 10.1016/j.jaci.2017.12.1007 [doi] (2018). 10.1016/j.jaci.2017.12.1007 29522843

[33] Xu M (2018). An interleukin-25-mediated autoregulatory circuit in keratinocytes plays a pivotal role in psoriatic skin inflammation. Immunity.

[34] Senra L (2016). Keratinocyte-derived IL-17E contributes to inflammation in psoriasis. J Invest Dermatol.

[35] Veldhoen M, Hocking RJ, Atkins CJ, Locksley RM, Stockinger B (2006). TGFbeta in the context of an inflammatory cytokine milieu supports de novo differentiation of IL-17-producing T cells. Immunity.

[36] Sonobe Y (2009). Interleukin-25 expressed by brain capillary endothelial cells maintains blood-brain barrier function in a protein kinase Cepsilon-dependent manner. J Biol Chem.

[37] Angkasekwinai P (2007). Interleukin 25 promotes the initiation of proallergic type 2 responses. J Exp Med.

[38] Suzukawa, M. *et al*. Epithelial cell-derived IL-25, but not Th17 cell-derived IL-17 or IL-17F, is crucial for murine asthma. *J Immunol*, **189**, 3641–3652, doi:jimmunol.1200461 [pii] 10.4049/jimmunol.1200461 [doi] (2012). 10.4049/jimmunol.1200461 PMC3812057 22942422

[39] Nakanishi W (2013). IL-33, but not IL-25, is crucial for the development of house dust mite antigen-induced allergic rhinitis. PLoS One.

[40] de Boer OJ (2010). Differential expression of interleukin-17 family cytokines in intact and complicated human atherosclerotic plaques. J Pathol.

[41] Hvid, M. *et al*. IL-25 in atopic dermatitis: a possible link between inflammation and skin barrier dysfunction? *J Invest Dermatol*, **131**, 150–157, doi:S0022-202X(15)34994-0 [pii] 10.1038/jid.2010.277 [doi] (2011). 10.1038/jid.2010.277 20861853

[42] Caruso R (2009). Interleukin-25 inhibits interleukin-12 production and Th1 cell-driven inflammation in the gut. Gastroenterology.

[43] Xu M, Dong C (2017). IL-25 in allergic inflammation. Immunol Rev.

[44] Wang W (2018). Bronchial Allergen Challenge of Patients with Atopic Asthma Triggers an Alarmin (IL-33, TSLP, and IL-25) Response in the Airways Epithelium and Submucosa. J Immunol.

[45] Schneider C, O’Leary CE, Locksley RM (2019). Regulation of immune responses by tuft cells. Nature reviews. Immunology.

[46] Van Dyken SJ (2014). Chitin activates parallel immune modules that direct distinct inflammatory responses via innate lymphoid type 2 and gammadelta T cells. Immunity.

[47] Senra L (2019). IL-17E (IL-25) Enhances Innate Immune Responses during Skin Inflammation. J Invest Dermatol.

[48] Fort, M. M. *et al*. IL-25 induces IL-4, IL-5, and IL-13 and Th2-associated pathologies *in vivo*. *Immunity*, **15**, 985–995, doi:S1074-7613(01)00243-6 [pii] (2001). 10.1016/s1074-7613(01)00243-6 11754819

[49] Yang D (2019). Dysregulated Lung Commensal Bacteria Drive Interleukin-17B Production to Promote Pulmonary Fibrosis through Their Outer Membrane Vesicles. Immunity.

[50] Horai R (1998). Production of Mice Deficient in Genes for Interleukin (IL)-1, IL-1, IL-1 /, and IL-1 Receptor Antagonist Shows that IL-1 Is Crucial in Turpentine-induced Fever Development and Glucocorticoid Secretion. J Exp Med.

[51] Ishii A (2010). Development of IL-17-mediated delayed-type hypersensitivity is not affected by down-regulation of IL-25 expression. Allergol Int.

